# Sportive lemurs elevate their metabolic rate during challenging seasons and do not enter regular heterothermy

**DOI:** 10.1093/conphys/coab075

**Published:** 2021-09-11

**Authors:** Janina Bethge, Jean Claude Razafimampiandra, Arne Wulff, Kathrin H Dausmann

**Affiliations:** 1 Functional Ecology, Institute of Zoology, Universität Hamburg, Martin-Luther-King-Platz 3, 20146 Hamburg, Germany; 2 Mention Zoologie et Biodiversité Animale, Faculté des Sciences, Université d’Antananarivo, B.P. 906, 101 Antananarivo, Madagascar

**Keywords:** Dry forest, energy budgets, Lepilemur edwardsi, Madagascar, primates, seasonality, thermoregulation

## Abstract

Animals experience seasonal changes of environmental and ecological conditions in most habitats. Fluctuations in ambient temperature have a strong influence on thermoregulation, particularly on small endothermic mammals. However, different mammalian species cope differently with these changes. Understanding the physiological responses of organisms to different seasons and analysing the mechanisms that account for intra- and inter-specific differences and the ecological consequences of these variations is important to predict species responses to climatic changes. Consequences of climatic changes will be most pronounced in climatically already challenging habitats, such as the dry regions of western Madagascar. We aimed to identify the seasonal responses and adaptive possibilities in energy budgeting of *Lepilemur edwardsi*, a small primate of this habitat, by measuring metabolic rate (MR; open-flow respiratory) and skin temperature in the field during different seasons. Resting metabolism was generally low, but our study did not detect any signs of regular heterothermic episodes, despite the fact that these are known in other sympatrically living lemurs with a similar lifestyle. Surprisingly, *L. edwardsi* responded by elevating its resting MR in the poor-resourced dry season, compared to the better-resourced wet season, presumably to master detoxification of their increasingly toxic diet. As body mass decreased over this time, this strategy is obviously not energetically balanced on the long term. This is cause for concern, as it suggests that *L. edwardsi* has a very small leeway to adjust to changing conditions as experienced due to climate change, as dry season are expected to become longer and hotter, straining water budgets and food quality even more. Moreover, our findings highlight the importance of studying physiological parameters directly in the field and under differing climatic conditions.

## Teaser

The Malagasy sportive lemur *Lepilemur edwardsi* shows a surprising response to the lean dry season. Instead of keeping energy expenditure generally low, it shows an increase while resting. As it loses weight, compensation does not seem to be fully balanced, which is concerning as longer dry periods are predicted with climate change.

## Introduction

Endothermic species regulate their body temperature (T_b_) mainly endogenously and independently of ambient temperature (T_a_). To cope with warm and dry habitats species often express strategies to save water and energy during the hottest and driest periods of the day or the year. For instance, by having a generally low metabolism (Lovegrove, 2000, 2003; Swanson *et al.*, 2017), which is sometimes accompanied by tolerance of high daily fluctuations of T_b_, as is the case in some rodents and camels from hot deserts (Schmidt-Nielsen *et al.*, 1981; Lovegrove and Heldmaier, 1994), and bats roosting in open vegetation at high T_a_ (Reher and Dausmann, 2021). This enables them to reduce their energy requirements and respiratory evaporative water loss (Schmidt-Nielsen *et al.*, 1957). Another response is to shift the thermoneutral zone, the range of T_a_ where basal metabolic rate (BMR) is sufficient to maintain normothermic T_b_ without any additional energetic costs, from a lower T_a_ (range in winter) to a higher T_a_ (range in summer) (Scholander, 1955; Heldmaier and Steinlechner, 1981). This shift is species specific and can occur seasonally and regionally and optimizes the zone of minimal energy expenditure to the prevailing conditions (Chamane and Downs, 2009; Kobbe *et al.*, 2014; Zhao *et al.*, 2014; Bethge *et al.*, 2017). Adapting to unpredictable changes and fluctuations of environmental conditions can be extremely challenging, making species highly vulnerable to additional anthropogenic changes in their habitats (Pörtner and Farrell, 2008; Khaliq *et al.*, 2014; Radchuk *et al.*, 2019). Due to their unfavourable surface-to-volume ratio, small endothermic animals (Kleiber, 1947; Hayssen and Lacy, 1985) are especially affected by varying and extreme T_a_ (Pörtner and Farrell, 2008). Some small mammals and birds even abandon endothermy and become heterothermic to survive lean seasons, even at comparatively high T_a_ (McKechnie and Mzilikazi, 2011; Dausmann *et al.*, 2012). Heterothermy describes physiological states of active depression of metabolic rate (MR) and thereby a controlled interruption of normothermia and almost all bodily functions, which can last from a few minutes (daily torpor) to many months (hibernation; Geiser, 2004; Heldmaier *et al.*, 2004; Ruf and Geiser, 2015; Reher and Dausmann, 2021). Small mammalian species that remain normothermic throughout the year often show a lower MR in dry and unpredictable habitats in comparison to species in habitats with more reliable resource availability (Speakman, 1999; Lovegrove, 2000). This is especially true for arboreal folivores that have a generally low metabolism, presumably due to their unfavourable diet that is comparatively low in nutrients and high in fibre and cellulose. Consistent with this low metabolism is the sedentary lifestyle and relatively low muscle mass of arboreal folivores (McNab, 1978a; Cork and Foley, 1991), e.g. in the three-toed sloth (*Bradypus variegatus*; Pauli *et al.*, 2016) or the slow loris (*Loris tardigradus*; Müller *et al.*, 1985).

Indeed, members of the arboreal, tropical and nocturnal Malagasy lemur family Lepilemuridae (e.g. *Lepilemur ruficaudatus* and *Lepilemur petteri*) show particularly low mass-specific resting MR (RMR; metabolism during inactivity; Schmid and Ganzhorn, 1996; Bethge *et al.*, 2017, published as *Lepilemur leucopus*). The only genus within this family, the sportive lemurs, are able to survive in even the most unpredictable and hottest habitats of Madagascar, from the dry south with its distinctive xerophytic spiny forest to the west with its deciduous dry forests and high degree of seasonality (Mittermeier *et al.*, 2010). Given the body size, lifestyle and diet of this lemur genus, it was previously hypothesized that sportive lemurs might become heterothermic to cope with lean seasons (Ganzhorn, 1988; Schmid and Ganzhorn, 1996). Moreover, members of a related lemur family, the Cheirogaleidae (*Cheirogaleus spp*. and *Microcebus spp*.) that live sympatrically with *Lepilemur spp*., undergo daily torpor and hibernation regularly as a seasonal response (Dausmann and Warnecke, 2016). However, up to now there are no scientifically confirmed cases of heterothemic episodes in the Lepilemuridae. *Lepilemur petteri*, living in the dry south of Madagascar, adapts seasonally by reducing its RMR in the hotter wet season and elevating its resting metabolism in the food constrained and more unpredictable dry season. Overall, this species seems to be more constraint by high T_a_, suggesting a small scope to tolerate changing environmental conditions.

*Lepilemur edwardsi* (800–1000 g; head–body length, ~27 cm), sister species of *L. petteri*, lives in less unpredictable, but also challenging conditions in the dry deciduous forests of western Madagascar (Mittermeier *et al.*, 2010), a region characterized by a pronounced seasonality. The wet, food-abundant season is characterized by an average T_a_ between 23°C and 36°C and a high yearly mean precipitation of 1562.5 mm (Randrianambinina *et al.*, 2007; Klein *et al.*, 2018). During the dry season there is no precipitation for ~6 months, and animals have to physiologically and behaviourally adapt to the accompanying scarcity of food and water together with slightly lower temperatures (T_a_ averages ~37°C during the day and 17°C during the night; Randrianambinina *et al.*, 2007). These dry season conditions are assumed to be extremely challenging for *L. edwardsi* due to its folivorous diet (Mittermeier *et al.*, 2010), as the food trees grow young high-quality leaves in the wet season, which decrease their protein content with leaf age and thus the ongoing dry season (Thalmann, 2001; Ganzhorn, 2002). *Lepilemur edwardsi* is pair-living and rests in tree holes during the day (Rasoloharijaona *et al.*, 2003; Mittermeier *et al.*, 2010). These resting sites seem to be an important resource for this species to shelter from climatic extremes, thus making the existing and ongoing habitat destruction due to climatic changes but also due to anthropogenic impacts, especially severe (Rasoloharijaona *et al.*, 2003; Wulff, 2020).

In this study, we investigated the physiological adaptations of *L. edwardsi* to seasonal changes in climatic conditions and food availability and quality, not least to allow for inferences on flexibility in the face of climate change and anthropogenic disturbances on this highly endangered species (Louis *et al.*, 2020). *Lepilemur edwardsi* lives farther north than *L. petteri*, in a different habitat with different climatic conditions, which is why we wanted to examine if the physiological adaptations found in *L. petteri* are a general trait of sportive lemurs or solely a specific adaptation to their respective habitats. In particular, we wanted to verify whether heterothermic episodes occur in *L. edwardsi* and if and how resting metabolism is adjusted throughout the season by measuring RMR and skin temperature (T_skin_) in the field under natural conditions and during different seasons.

## Materials and methods

### Study site

We conducted the study around the Ampijoroa Forest Station within the Ankarafantsika National Park (S 16° 19′, E 46° 48′) in Jardin Botanique A, a 30.6-ha research area with dry deciduous forest in western Madagascar. The region is characterized by a distinct wet season from November to April and a food constraint dry season from May to October. Temperatures are generally high with minimal T_a_ ~23°C in the wet and 17°C in the dry season, and daily maximal T_a_ ~36°C and 37°C, respectively, with almost no precipitation for up to 6 months (Randrianambinina *et al.*, 2003, 2007).

### Animal captures

We captured 27 individuals of *L. edwardsi* during three sampling periods in 2018 and 2019. We measured the RMR during the wet season to get the baseline RMR, when resources are abundant and T_a_ is relatively stable. During the early dry season, individuals have to respond to the beginning of the deteriorating environmental conditions and food availability. Whereas, during the late dry season individuals have to cope with the full extent of strongly decreased food availability and the ongoing lack of rainfall. A total of 11 individuals (9 males, 2 females) were captured during the wet season (January–February 2018), 12 individuals (6 males, 6 females) during the early dry season (May–July 2019) and 17 individuals (7 males, 10 females) during the late dry season (August–October 2018). We located the individuals in their sleeping sites in the mornings (09:00–12:00 hours), captured them by hand and anaesthetized them with 0.1 ml/kg ketamine hydrochloride (Ketamidor ® 100 mg/ml, WDT, Garbsen, Germany). Afterwards, we weighed the individuals to the nearest 1 g, sexed and individually marked them with a subcutaneously injected passive integrated transponder (Trovan, EURO I.D. Usling GmbH, Weilerswist, Germany). Individuals were released after the measurements of RMR at their trapping locations at dusk the next day. Some individuals were used in more than one sampling period. This is accounted for in the statistical models.

### Measurements of MR

MR was determined by measuring oxygen consumption using open-flow respirometry in pull mode with a portable oxygen analyser (OxBox, designed and constructed by T. Ruf & T. Paumann, FIWI, University of Veterinary Medicine Vienna, Austria) with chemo-electric oxygen sensors (Bieler & Lang, Achern, Germany; for more details, see Dausmann *et al.*, 2009) powered by a 12-V (100 Ah) battery. The oxygen sensor was calibrated immediately before and after each field trip in the laboratory with calibration gas prepared by a gas-mixing pump [20.95%, 20.32% and 19.90% atmospheric oxygen (O_2_); type 2KM300/a; H. Wösthoff Messtechnik GmbH, Bochum, Germany]. A closed box served as respiratory chamber (30 cm × 25 cm × 23 cm). It was connected to the oxygen analyser with airtight tubes (Tygon, Saint-Gobain, Courbevoie, France) and had an air inlet on the opposite side of the box (scheme of the setup: Suppl. Fig. 1). Measurements started in the mornings (around 09:30–12:00 hours, depending on capture time) and were stopped after ~24 hours, thus providing continuous measurements over this time. We conducted the measurements directly in the field under naturally fluctuating daily T_a_. All animals were constantly monitored to ensure wellbeing and calm behaviour. Generally, individuals remained calm during the measurements. In the few cases where animals seemed stressed, the measurements were discontinued and they were immediately returned to their capture sites. Airflow was set at 70–100 l/h, which has proved appropriate for our set-up in many studies (e.g. Turner *et al.*, 2017), matching the size of the respiratory chamber and adequate wash-out rates and was constantly monitored and regulated by the flowmeter of the oxygen analyser (OxBox). The sample air was dried with silica gel and filtered before entering the flowmeter, subsampling and oxygen analysis, and oxygen content was determined once per minute. Reference air (ambient air from the environment next to the respiratory chamber) was also dried with silica gel and filtered before entering the analyser and analysed for 5 minutes every hour to account for drift of the oxygen sensors (Suppl. Fig. 1). We did not scrub the incurrent air into the respiratory chamber of water vapour, as we wanted to measure natural responses to prevailing conditions and avoid physiological stress by being exposed to completely dry air. Volumes were corrected for standard conditions for temperature and pressure, with barometer pressure provided by a nearby weather station and temperature of the airflow measured by the flowmeter of the oxygen analyser (OxBox). When CO_2_ is not scrubbed from the excurrent sample air or measured, as we only had access to silica gel in the field, we can assume a value based on the volume of O_2_ (volO_2_) and substitute the volume of CO_2_ by using the respiratory quotient (RQ) of volO_2_ (Lighton, 2008). Hence, the rate of oxygen consumption (V̇O_2_) was calculated as $\mathrm{ml}\ {\mathrm{O}}_2{\mathrm{h}}^{-1}$with the equation given by Lighton (2008) specifically for this set-up:$\dot{V}{\mathrm{O}}_2=\frac{{\mathrm{F}\mathrm{R}}_{\mathrm{e}}({\mathrm{F}}_{\mathrm{i}}{\mathrm{O}}_2-\mathrm{F}{\hbox{'}}_{\mathrm{e}}{\mathrm{O}}_2)}{[1-{\mathrm{F}}_{\mathrm{i}}{\mathrm{O}}_2(1-\mathrm{RQ})]}$, where ${\mathrm{FR}}_{\mathrm{e}}$ is the excurrent flow rate and $({\mathrm{F}}_{\mathrm{i}}{\mathrm{O}}_2-{{\mathrm{F}}^{\hbox{'}}}_{\mathrm{e}}{\mathrm{O}}_2)$ gives the difference in fractional concentration of oxygen entering and exiting the respiratory chamber. When assuming an RQ of 0.85 (oxidation of ~50% carbohydrate and 50% fat; see below), the maximum error amounts approximately to 3% (Koteja, 1996, Lighton, 2008), which is acceptable for our study, as we are interested in patterns and adaptations. Additionally, we scrubbed most of the water from the sample and reference air indicated by F’eO_2_ (Lighton, 2008). The data of the oxygen analyser were analysed using ClampFit v10.3.1.4 (Molecular Devices, Sunnyvale, USA); see Dausmann (2009) for details. To calculate mass-specific RMR, we divided RMR by body mass $(\mathrm{ml}\ {\mathrm{O}}_2\ {\mathrm{h}}^{-1}{\mathrm{g}}^{-1})$; all RMR data is given as mass-specific RMR.

**Figure 1:**
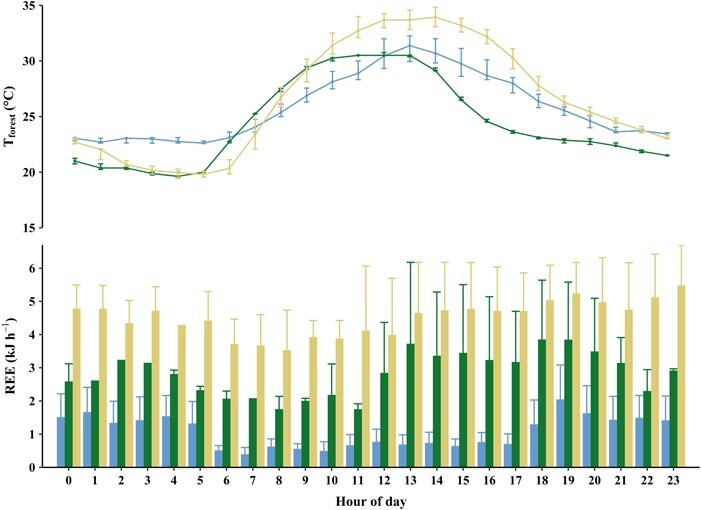
REE of *L. edwardsi* and daily fluctuations of ambient forest temperature during the three sampling periods. Hourly REE of *L. edwardsi* (wet season, *N* = 11; early dry season, *N* = 12; late dry season, *N* = 17) and median hourly ambient forest temperature (T_forest_) in the wet season (blue columns; blue line), the early dry season (green columns; green line) and the late dry season (yellow columns; yellow line) in the dry forest of Ampijoroa. The whiskers of the REE show the standard deviation; and the median T_forest_ whiskers show hourly maximal T_forest_ and minimal T_forest_.

### Temperature measurements

Shortly before the RMR measurements we measured the rectal temperature (T_rectal_) of the individuals as a proxy of core body temperature with a handheld thermometer (resolution: ±0.1°C, MT1P21 flexible, Microlife AG, Widnau, Switzerland). During respirometry, T_skin_ of the animals was recorded to the nearest 0.5°C once per minute using a temperature data logger (custom made by J. Sannert, Hamburg, Germany) mounted on a collar with the temperature sensor on the inside in direct contact with the skin (4 g) Although T_skin_ does not precisely represent core T_b_, numerous studies have shown that it gives a valid (and less invasive) proxy to gain insights into general patterns of T_b_ regulation in small mammals, especially during resting phases, when possible torpor episodes would be expected (Dausmann, 2005; Langer and Fietz, 2014). Directly before release of the individuals, we equipped the majority of the individuals with a temperature data logger (Thermochron iButton, Maxim Integrated Products, San Jose, USA) glued to the inside of a radio transmitter collar (total weight, 12.5 g; PIP3 longlife tag, Biotrack Ltd, Dorset, UK) to measure long-term T_skin_ (wet season, *N* = 2; early dry season, *N* = 7; late dry season, *N* = 12) every 30 minutes. At the end of the respective sampling period, we recaptured these individuals via radio tracking and recovered the data loggers. The duration of these T_skin_ measurements ranged from 12 to 39 days, depending on the day of respirometry. We defined daily torpor episodes to occur if T_skin_ decreased below 25°C for at least 1 hour (following the definition of Kobbe *et al.*, 2011), which is clearly below the regular circadian rhythm, with MR decreasing concordantly (if occurring during RMR measurements). We are aware that there might be an influence of environmental heat load on T_skin_, and therefore chose the threshold for torpor episodes with a corresponding margin.

During respirometry, the temperature in the respiratory chamber (T_chamber_) was recorded simultaneously once per minute with data loggers (Thermochron iButton) placed inside the respiratory chamber. Additional temperature loggers (Thermochron iButton) were placed inside and outside (on the shady side) of tree holes (wet season, *N* = 13; early dry season, *N* = 5; late dry season, *N* = 11) where *L. edwardsi* were known to rest during the day in the dry forest, which recorded temperature in the forest (T_forest_) and in the treehole (T_treehole_) every 30 minutes.

### Data analysis and statistics

Data were analysed using Cran R (R Core Team, 2019) and the packages ‘plyr’ (Wickham, 2011), ‘dplyr’ (Wickham *et al.*, 2019), ‘lubridate’(Grolemund and Wickham, 2011), ‘ggplot2’ (Wickham, 2016), ‘mgcViz’ (Fasiolo *et al.*, 2020) and ‘mgcv’ (Wood, 2017).

We only included the lowest 30% of the continuous daily and nightly RMR data of each individual in the analyses to exclude any phases of short stress, disturbance or activity of the individuals (Bethge *et al.*, 2017). This is a conservative approach to ensure only resting values are considered. To control for autocorrelation, we calculated hourly means per individual per season and assigned these to the average T_chamber_ during this hour. We wanted to examine the influence of different variables on the RMR (ml O_2_ h^−1^ g^−1^) of *L. edwardsi* and model this functional relationship. As the visual data exploration showed a non-linear relationship between the explanatory variables (T_a_, month, sex) and the response variable (RMR) we chose a generalized additive mixed model (GAMM) to estimate smooth functional relationships between theses variables (Zuur *et al.*, 2009; Wood, 2017). The pACF-plots still showed a slight autocorrelation in the data, thus we controlled for autocorrelation using different approaches: firstly, we included time as a random effect; secondly, we implemented an autoregressive structure [autoregressive model (AR1), autoregressive-moving-average model (ARMA)] in the GAMM; and thirdly, we used a combination of both approaches (Suppl. Table1). We selected the ‘best model’ based on Akaike’s information criterion (AIC) and a visual approach using the gam.check()-function of the package ‘mgcv’ (Zuur *et al.*, 2009; Wood, 2017; Pedersen *et al.*, 2019). We modelled 12 GAMMs with different explanatory variables and random effects and incorporated an autoregressive structure into these models (AR1, ARMA). The GAMM with time as a random effect performed better than the models with the autoregressive structure, showed a lower AIC and explained 64.3% of the deviance. Hence, our final model included smoother for T_chamber_ (cubic regression spline smoother), month as a proxy for season (cyclic cubic spline smoother), a smoother for the interaction of T_chamber_ and month and sex (two-level categorical variable; Table 3). Since some individuals were measured in multiple seasons, we included ID and time as random effects and used restricted maximum likelihood instead of cross-validation criteria for smoothing parameter selection (following the recommendation of Wood, 2011).

Since the data were non-independent (some individuals were measured more than once in different seasons) and showed non-normality (Shapiro–Wilk Test: *P* < 0.001), we applied an unpaired Wilcoxon rank sum test with a Bonferroni correction to test for differences between the body mass and the minimal and maximal T_skin_ of all individuals between the seasons. As the wet season sample size for T_skin_ was relatively small (*N* = 2), the statistical comparisons between the wet season and the two sampling periods during the dry season, should be interpreted cautiously but nevertheless gives a good impression of the overall seasonal adaptations in thermoregulation of *L. edwardsi*.

We calculated energy expenditure (REE; kJ h^−1^) and daily resting energy expenditure (DREE; kJ 24 h^−1^) under field conditions assuming an average RQ of 0.85 (Dausmann *et al.*, 2009) and thus an oxycaloric equivalent of 20.37 kJ/L O_2_ (Schmidt-Nielsen, 1997). To interrelate these data to the environmental conditions, average T_forest_ (natural habitat) for each hour of the day was calculated for each sampling period separately (wet season, early dry season and late dry season) to account for the daily rise and fall in temperature, and the RMR determined from the respiratory measurements during this sampling period for this particular temperature was appointed. For DREE the values for each hour throughout the day were added.

Additionally, we calculated the mean energy expenditure of the lowest 30% of the RMR data for each individual during each season to compare our results with data from other primates by using the mass-specific BMR scaling equation for primates provided by McNab (2008) [BMR (kJ h^−1^) = 0.037 × body mass^0.792^]. In our field set-up, we did not meet all the requirements for BMR measurements; however, we are confident that the lowest RMR-values approximate BMR as individuals were resting during the day and the last food intake had been several hours before.

## Results

The median of the minimal T_forest_ during the wet season amounted to 22.9°C during the night and the median maximal T_forest_ to 29.1°C in the middle of the day. T_forest_ in the early dry season fluctuated between the median minimal T_forest_ of 20.75°C during the night and maximal median T_forest_ of 27.5°C during the day. During the late dry season T_forest_ fluctuated more strongly with a minimal median T_forest_ of 22.5°C; however, maximal median T_forest_ was higher with 32.5°C during the daytime (Fig. 1). The mean body mass of males and females did not differ significantly between the sexes in any of the different sampling periods. Mean body mass of both sexes in the wet season amounted to 827 ± 84 g (*N* = 11) and was significantly higher in the early dry season with a mean body mass of 929 ± 104 g (*N* = 12; Wilcoxon rank sum test; wet season vs. early dry season, *P* = < 0.001). Mean body mass in the late dry season was 795 ± 111 g (*N* = 12) and was significantly lower than mean body mass in the wet season (Wilcoxon rank sum test; wet season vs. late dry season, *P* = < 0.001). Furthermore, individuals lost weight during the dry season, as mean body mass in the early dry season was significantly higher than in the late dry season (Wilcoxon rank sum test; early dry season vs. late dry season, *P* = < 0.001). Five females were probably pregnant in the late dry season with a mean body mass of 1049 ± 32 g.

### Skin temperature of free-ranging *L. edwardsi*

**Figure 2:**
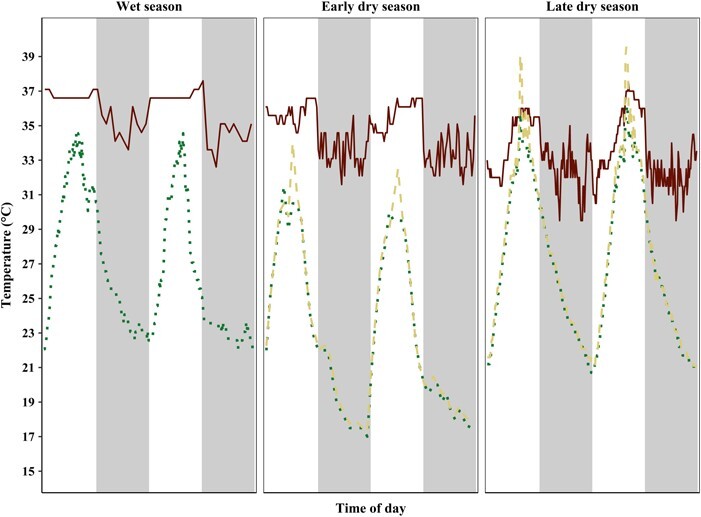
Fluctuations of resting site temperatures and of skin temperature of *L. edwardsi* during the three sampling periods. Skin temperature (red, solid line) and ambient temperature outside (green, dotted line) and inside (yellow, dashed line) of the resting site of the same individual of *L. edwardsi* on two consecutive days during the wet season, early dry season and late dry season. Grey blocks show the dark phase and hence phase of activity of *L. edwardsi*.

The long-term field T_skin_ measurements of all individuals showed strong fluctuations ranging from 31.1°C to 39.6°C in the wet season (*N* = 2), from 27.6°C to 41.1°C in the early dry season (*N* = 7) and from 28.0°C to 40.1°C (*N* = 12) in the late dry season. During the wet season, individuals reached a maximal mean T_skin_ of 36°C between 12:00 and 17:00 hours when mean T_forest_ was highest (26.6–29.5°C). Minimal mean T_skin_ of 34.4°C was reached at 04:00 hours when mean T_forest_ was 21.6°C during their active phase at night. Mean T_skin_ in the early dry season was highest between 12:00 and 17:00 hours with 36°C at a mean T_forest_ between 23.7°C (17:00 hours) and 29.2°C (12:00 hours) and lowest at 04:00 hours with a minimal mean T_skin_ of 33.8°C and a mean T_forest_ of 18.1°C. In the late dry season individuals had a maximal mean T_skin_ of 37°C between 14:00 and 17:00 hours at a mean T_forest_ between 28.8°C (17:00 hours) and 32.2°C (14:00 hours). During the night *L. edwardsi* showed a minimal mean T_skin_ of 33.6°C between 03:00 and 04:00 hours at a mean T_forest_ of 19°C. Thus, maximal T_skin_ of all individuals during all days of measurement in the wet season did not differ from the maximal T_skin_ in the early dry season, but was significantly lower than in the late dry season (Wilcoxon rank sum test; wet season vs. early dry season, *P* = 0.46; wet season vs. late dry season, *P* = 0.009). Whereas maximal T_skin_ during the late dry season was significantly higher than in the early dry season (Wilcoxon rank sum test; late vs. early dry season, *P* = < 0.001). Minimal T_skin_ of all individuals and all days of measurement in the two sampling periods of the dry season did not differ from each other (Wilcoxon rank sum test; late vs. early dry season, *P* = 0.27). However, minimal T_skin_ in the wet season was significantly higher than during both sampling periods of the dry season (Wilcoxon rank sum test; wet season vs. early dry season, *P* = < 0.001; wet season vs. late dry season, *P* = 0.001) and followed the daily T_a_ fluctuations. Overall, T_skin_ fluctuated more during the two sampling periods in the dry season, whereas T_skin_ in the wet season did not drop below 31°C (Fig. 2). T_skin_ dropped a few degrees at the beginning of the daily activity period at dusk (around 18:00 hours) with decreasing T_forest_ during all seasons and ranged at lower levels during the night while the individuals were active. This might be due to an increased influence of T_a_ on the T_skin_ logger when the animals are not curled up during activity. Nevertheless, as *L. edwardsi* rests in quite narrow tree holes during the day, we expect T_skin_ to approximate T_b_ during rest. At dawn (around 06:00 hours), T_skin_ increased again continuously with increasing T_forest_ resp. T_treehole_. Particularly, during the late dry season *L. edwardsi* seems to take advantage of the increasing daytime T_forest_ for passive uptake of external heat (resp. T_treehole_; Fig. 2).

### Seasonal differences in RMR and T_skin_

**Figure 3:**
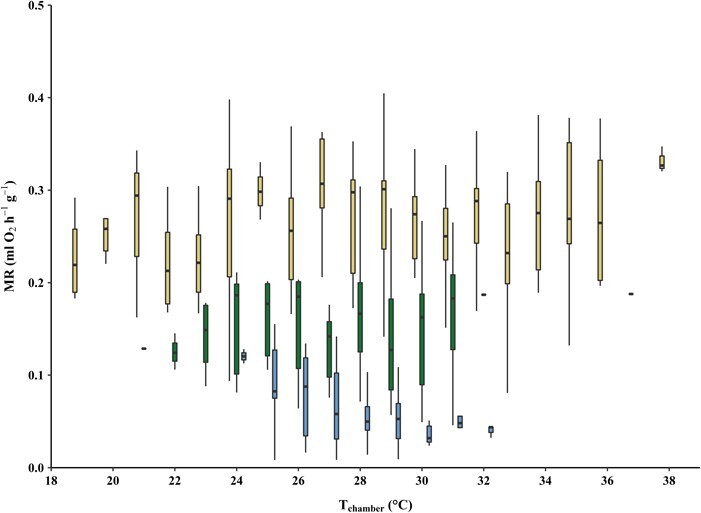
Seasonal differences in RMR of *L. edwardsi* at different ambient temperatures (respiratory chamber). RMRs of *L. edwardsi* at different ambient chamber temperatures (T_chamber_) during the wet season (blue; *N* = 11), early dry season (green; *N* = 12) and late dry season (yellow; *N* = 17). The boxplots show medians and quartiles, whiskers show maxima and minima.

**Figure 4:**
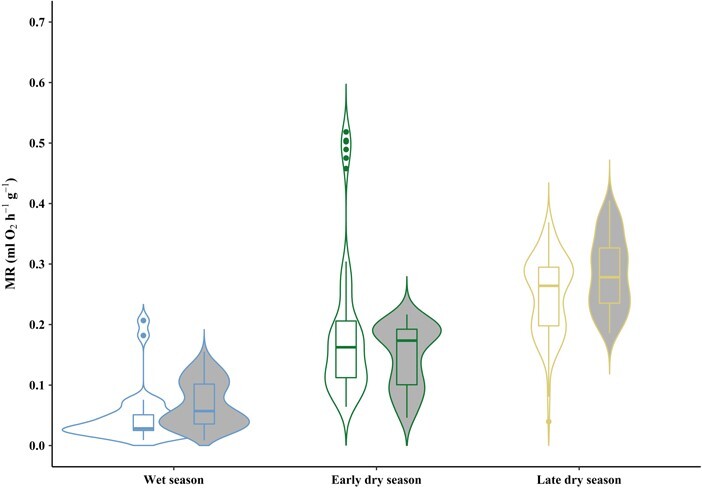
RMR of males and females of *L. edwardsi* during the three sampling periods. RMRs of males (white; wet season, *N* = 9; early dry season, *N* = 6; late dry season, *N* = 7) and females (grey; wet season, *N* = 2; early dry season, *N* = 6; late dry season, *N* = 10) of *L. edwardsi* during the wet season (blue; *N* = 11), early dry season (green; *N* = 12) and late dry season (yellow; *N* = 17). The boxplots show medians and quartiles; whiskers show maxima and minima. Violin shapes illustrate the distribution and density of the data.

There was no evidence of torpor episodes in any of the individuals during the wet and the early dry season as T_skin_ never dropped below 25°C (Table 1), neither during the RMR measurements, nor in the free-ranging individuals. However, in the late dry season one individual (Lepilemur 15) showed a sudden drop of T_skin_ from 31°C to 26°C at around 6:00 hours and a T_chamber_ of 19°C, followed by a distinct drop in RMR during the measurements, while being curled up in a resting position (Suppl. Fig. 2). T_rectal_ of this individual was below 33°C at the end of the RMR measurement (unfortunately, the handheld thermometer could not measure temperatures below 33°C). Generally, T_skin_ during the RMR measurements averaged around 36.1 ± 0.8°C (*N* = 11) in the wet season, 36.8 ± 0.8°C (*N* = 12) in the early dry season and 35.4 ± 2.1°C (*N* = 17) in the late dry season (Table 1). Whereas T_chamber_ fluctuated between 24.6–31.1°C in the wet season, 21.6–32.0°C in the early dry season and 19.1–40.1°C in the late dry season. T_rectal_ measured right before the beginning of the RMR-measurements (with an approximate time lag of <2 minutes before the first T_skin_ measurement), did not differ from the first data point of the T_skin_ measurements of all individuals during respirometry (Pearson’s Chi-squared test; *P* = 0.31). At the same T_chamber_, individuals had the lowest mean MR in the wet season (0.06 ± 0.04 ml O_2_ h^−1^ g^−1^), intermediate mean RMR in the early dry season (0.17 ± 0.10 ml O_2_ h^−1^ g^−1^) and the highest mean RMR in the late dry season (0.25 ± 0.07 ml O_2_ h^−1^ g^−1^; Figs 3 and 4).

**Table 1:** Individuals of *L. edwardsi* measured in the wet season, early dry season and late dry season; sex of the individuals (m = male, f = female); body mass (BM); mean skin temperature (T_skin_); rectal temperature (T_rectal_) of the individuals before the MR measurements; and mean MR for each season

		Wet season 2018					Early dry season 2019					Late dry season 2018				
Ind.	Sex	BM (g)	Mean T_skin_ (°C)	T_rectal_ (°C)	Mean MR (ml O_2_ h^−1^ g^−1^)	Mean MR (J^1^ h^1^ g^−1^)	BM (g)	Mean T_skin_ (°C)	T_rectal_ (°C)	Mean MR (ml O_2_ h^−1^ g^−1^)	Mean MR (J^1^ h^1^ g-1)	BM (g)	Mean T_skin_ (°C)	T_rectal_ (°C)	Mean MR (ml O_2_ h^−1^ g^−1^)	Mean MR (J^1^ h^1^ g^−1^)
1	m	671	36.0 ± 0.7	36.4	0.04 ± 0.02	0.89 ± 0.45						775	37.0 ± 0.2	36.8	0.29 ± 0.03	5.88 ± 0.67
2	m	877	36.4 ± 0.8	36.4	0.08 ± 0.04	1.69 ± 0.88										
3	m	947	35.9 ± 0.3	36.7	0.06 ± 0.03	1.29 ± 0.67	954	36.2 ± 0.6	36.3	0.09 ± 0.02	2.01 ± 0.42					
4	m	798	36.0 ± 0.6	34.5	0.08 ± 0.04	1.65 ± 0.84										
5	f											996	NA	34.9	0.33 ± 0.03	6.70 ± 0.55
6	m	744	36.4 ± 0.3	35.7	0.07 ± 0.03	1.48 ± 0.58										
7	m	836	NA	35.9	0.07 ± 0.03	1.59 ± 0.69						880	NA	37.2	0.28 ± 0.03	5.76 ± 0.63
8	m	910	36.5 ± 0.9	35.0	0.05 ± 0.03	1.04 ± 0.58						952	37.2 ± 0.4	36.8	0.21 ± 0.02	4.19 ± 0.43
9	m	965	35.2 ± 1.1	35.1	0.02 ± 0.01	0.43 ± 0.24						862	NA	35.6	0.28 ± 0.02	4.43 ± 0.39
10	f						946	36.8 ± 0.4	36.4	0.17 ± 0.02	3.61 ± 0.41	1064	37.1 ± 1.1	38.2	0.25 ± 0.07	5.00 ± 1.40
11	m	944	35.9 ± 0.5	35.3	0.08 ± 0.03	1.66 ± 0.57	934	36.5 ± 0.5	35.8	0.15 ± 0.02	3.16 ± 0.44	914	NA	38.3	0.33 ± 0.02	6.62 ± 0.41
12	f	747	36.4 ± 0.4	36.4	0.02 ± 0.01	0.48 ± 0.27	809	35.8 ± 0.7	35.2	0.49 ± 0.02	9.97 ± 0.48					
14	f	864	36.6 ± 0.3	35.9	0.04 ± 0.02	0.89 ± 0.45						911	35.7 ± 0.1	37.1	0.24 ± 0.03	4.88 ± 0.59
15	f											655	32.3 ± 2.9	35.0	0.22 ± 0.08	4.49 ± 1.56
16	f											684	37.3 ± 0.2	37.4	0.22 ± 0.05	4.42 ± 0.92
17	m						943	37.0 ± 0.4	37.1	0.07 ± 0.02	1.40 ± 0.45	887	37.5 ± 0.6	38.0	0.31 ± 0.09	6.41 ± 1.82
18	f											796	35.6 ± 1.4	35.6	0.26 ± 0.05	5.31 ± 0.94
19	f						938	37.0 ± 0.4	35.7	0.20 ± 0.03	3.98 ± 0.70	1047	34.5 ± 1.1	38.4	0.17 ± 0.03	3.55 ± 0.64
20	f											594	36.9 ± 0.5	38.0	0.29 ± 0.02	5.83 ± 0.45
21	f											1084	34.8 ± 0.9	36.4	0.19 ± 0.04	3.82 ± 0.89
22	m											801	35.4 ± 1.6	37.1	0.30 ± 0.06	6.14 ± 1.20
23	f						981	37.3 ± 0.3	NA	0.27 ± 0.02	5.43 ± 0.45	1066	37.1 ± 0.5	35.7	0.26 ± 0.02	5.30 ± 0.35
26	f						1136	NA	35.8	0.10 ± 0.02	1.95 ± 0.40					
28	f						814	NA	35.8	0.14 ± 0.03	2.91 ± 0.60					
31	m						898	37.3 ± 0.4	34.6	0.19 ± 0.01	3.88 ± 0.20					
32	m						858	36.6 ± 0.2	35.8	0.18 ± 0.01	3.74 ± 0.26					
33	m						819	37.8 ± 0.9	35.6	0.20 ± 0.01	4.03 ± 0.30					

During some of the T_skin_ measurements, the T_skin_-logger failed, given as NA.

To examine the seasonal influence and the influence of ambient T_chamber_ on the RMR of *L. edwardsi* we modelled the data with a GAMM, which showed an approximate significant influence of all smooth terms, i.e. explanatory variables (T_chamber_, month) on the response variable (RMR), indicating a high influence of month, i.e. season and T_chamber_ on the RMR of *L. edwardsi.*

### Daily resting energy expenditure

After calculating the ‘typical’ temperature profile for a day in each sampling period with T_forest_ (average temperature for each hour), we calculated the average DREE of *L. edwardsi* for each sampling period with the RMR determined in the respiratory measurements for this temperature and sampling period. Hourly resting energy expenditure (REE) during the early dry season was more varied than in the wet and late dry season, particularly during the hottest time of the day (Fig. 1). *Lepilemur edwardsi* showed a comparatively low DREE in the wet season of 25.1 ± 16.22 kJ 24 h^−1^, which increased more than 3-fold in the early dry season to 75.56 ± 39.50 kJ 24 h^−1^. DREE in the late dry season was even higher, almost 5-fold that of the wet season and 1.5-fold that of the early dry season with a DREE of 106.61 ± 30.65 kJ 24 h^−1^ (Table 2).

**Table 2:** Mean DREE, mean REE per hour and mean daily oxygen consumption, i.e. MR and mean mass-specific MR of *L. edwardsi* during the different seasons

Season	Mean DREE (kJ 24 h^−1^)	Mean REE (J^1^ h^1^ g^−1^)	Mean MR (ml O_2_ 24h^−1^)	Mean MR (ml O_2_ h^−1^ g^−1^)
Wet season 2018	25.1 ± 16.22	1.25 ± 0.78	1232.24 ± 796.3	0.06 ± 0.04
Early dry season 2019	75.56 ± 39.50	3.49 ± 2.05	3709.36 ± 1939.28	0.17 ± 0.10
Late dry season 2018	106.61 ± 30.65	5.13 ± 1.36	5233.5 ± 1504.87	0.25 ± 0.07

The mean energy expenditure of *L. edwardsi* amounted to 1.05 ± 0.42 kJ h^−1^ in the wet season, to 3.44 ± 1.78 kJ h^−1^ in the early dry season and 4.57 ± 1.07 kJ h^−1^ in the late dry season. Thus, accounting for ~approximately 15%, 41% and 58% of the mass-specific BMR value expected by the scaling equation in the wet season, early dry season and late dry season, respectively.

## Discussion

Our measurements of the RMR of the Malagasy primate species *L. edwardsi* showed that this species has to increase its generally low mass-specific RMR to cope with seasonally changing conditions, from the food abundant wet season to the scarce dry season.

### Low metabolism allows for a folivorous, potentially toxic, diet

By adopting a mainly sedentary lifestyle and a generally low metabolism, folivores living in dry habitats with unfavourable conditions (e.g. high seasonality, unpredictability of precipitation, etc.) are able to save energy and particularly water (Lovegrove, 2000, 2003; McNab, 2008; Swanson *et al.*, 2017). However, daily and seasonally fluctuating T_a_ can be a key physiological stressor, which can affect many aspects of physiology, most evidently those concerning thermoregulation, but also less obvious ones, such as hormonal responses (e.g. Weingrill *et al.*, 2004; Cristóbal-Azkarate *et al.*, 2016). Our study showed that the RMR of the small folivorous *L. edwardsi* was significantly influenced by T_a_ as well as generally by season and presumably by the associated changes in food and water availability. Basal energy expenditure of *L. edwardsi* during the wet season was extremely low (1.05 ± 0.42 kJ h^−1^; Table 2), matching the generally low resting metabolism of other roughly same-sized sportive lemur species: *L. petteri* (Bethge *et al.*, 2017) and *L. ruficaudatus* (Schmid and Ganzhorn, 1996). This value is much lower (~15% of the expected value) than the value predicted based on the BMR scaling equation for primates (McNab, 2008), which would be 7.63 kJ h^−1^ for primates of comparable body mass. However, these allometric scaling equations are widely debated (see, for example Kozlowski and Konarzewski, 2004; Glazier, 2005; White *et al.*, 2007) as they often do not account for ecological factors of the species, such as home range sizes (Nunn and Barton, 2000). During the wet season, temperatures are quite benign in Ampijoroa and daily fluctuations in T_a_ are comparatively small. Furthermore, precipitation is highest during this time of the year and food is abundant. Low metabolism might be essential for a folivorous diet, due to its relatively low quality and, in the case of sportive lemurs, toxicity (Ravelomandrato, 2006). To minimize the need to invest in detoxification, sportive lemurs might decrease their general food intake, which is only possible when MR is low (McNab, 1978b; Moore *et al.*, 2015) and energetic investments (e. g., behaviour, reproduction) are well economized. *Lepilemur edwardsi* is possibly able to subsist on this extremely low resting metabolism by decreasing general activity and maintaining only comparatively small home ranges (Warren and Crompton, 1997).

**Table 3:** Results of the final GAMM with MR as response variable: types of smooth terms, maximum possible degrees of freedom for the smooth terms (*k’*), effective degrees of freedom (EDF), index for pattern in the residuals (*k*-index) and *P*-value (calculated, based on distribution of *k*-index after randomizing order of residuals) for each explanatory variable.

Explanatory variables	Smooth terms	*k’*	EDF	*k*-index	*P*-value
Ambient chamber temperature (T_chamber_)	Cubic regression spline	19	4.480	0.59	0.000817^***^
Interaction term T_chamber_: Month	Cyclic cubic regression spline	19	3.397	0.59	<0.001^***^
Month	Cyclic cubic regression spline	4	2.202	0.22	<0.001^***^
Individuals	Random effect	1	0.773	0.24	0.0238
Time	Random effect	1	0.898	0.85	0.0013^**^
Sex	Factor	-	-	-	0.453

^*^P = 0.01.

^**^P = 0.001.

^***^P < 0.001.

It was previously hypothesized that sportive lemurs use heterothermic responses, such as daily torpor (Ganzhorn, 1988; Schmid and Ganzhorn, 1996), to counter increased energy and water demands during the challenging dry seasons of Madagascar. However, during our study we could not detect regular employment of heterothermy. We only observed one individual showing such a physiological response (Lepilemur 15): a sudden drop in RMR and T_skin_ (as low as 26°C; Suppl. Fig. 2). The lack of a comparable drop in other individuals suggests that maybe this ‘torpor-like’ episode was not a regular seasonal response, but rather possibly an emergency response to low body condition, T_a_ and food availability, as it has been observed in the African bushbaby *Galago moholi* and the least gerbil *Gerbillus pusillus* (Buffenstein, 1985; Nowack *et al.*, 2013). In *G. moholi* only subadult individuals with poor body condition entered daily torpor on rare occasions and individuals that showed a T_skin_ ≤ 19°C had problems to return to normothermy without external, passive heating by T_a_ (Nowack *et al.*, 2013). In the case of *G. pusillus*, food- and water-deprived individuals increased their torpor frequency and a critical T_a_ threshold during torpor was reached below 15°C (Buffenstein, 1985)*.* The observed individual of *L. edwardsi* also had a comparatively low body mass (655 g; Table 1) and was probably a sub-adult offspring from the previous breeding season. Entry into torpor commenced towards the end of the measurement (between 06:00 and 07:00 hours) when the individual had already been deprived of food for ~24 hours. As starvation has an effect on the use of heterothermy (Morhardt and Hudson, 1966; Buffenstein, 1985; Bozinovic *et al.*, 2007), this could have been the trigger for this ‘torpor-like’ episode. 

### Increased metabolism during the challenging dry season presumably allows for detoxification of low-quality food, while securing adequate water uptake

Compared to the wet season, individuals increased their resting metabolism during the dry season conditions independently of the changes in T_a_ (Fig. 3), especially at the end of the dry season, which probably is the harshest and most challenging time of the year for survival, as also evidenced by the significant weight loss of the individuals during this time. Over the course of the year, the RMR of *L. edwardsi* tripled from the wet to the early dry season and increased approximately 5-fold from the wet to the late dry season, even at the same T_a_ (Table 2; Figs 1 and 3). This is analogous to the pattern found in *L. petteri*; this species was also found to increase its RMR in the dry season presumably due to increased detoxification efforts of its potentially toxic diet and the hot daytime temperatures during the dry season of southern Madagascar. During the wet season, when food is abundant, *L. petteri* has a much lower RMR and is presumably able to compensate the increased heat production by its sedentary behaviour. However, due to cooler nights during the dry season in the South, *L. petteri* needs more energy to thermoregulate, requiring an increased foraging effort. This in turn results in a higher need for detoxification, which is negatively influenced by the high daytime T_a_ during this season (Bethge *et al.*, 2017). Similarly, *L. edwardsi* also seems to be more affected by the effect of the hot daytime temperatures towards the end of the lean dry season on their food quality, than low nighttime temperatures. As *L. edwardsi* shows an elevated resting metabolism even at temperatures that also occur during the wet season, thermoregulation cannot be the only factor inducing this increase in resting metabolism during the (late) dry season (Figs 1 and 3). However, as the weight loss of *L. edwardsi* is presumably mostly due to the loss of metabolic inactive, white adipose tissue, this could also contribute to an apparent increase in mass-specific RMR.

Many plants adjust their nutritional content (e.g. protein) and their plant secondary metabolites (PSMs), depending on leaf age (Coley, 1983), herbivore defence (Mithöfer and Boland, 2012) and T_a_ (Ganzhorn and Wright, 1994; Ganzhorn, 1995; Dearing, 2013). Moreover, a folivorous diet is generally characterized by a low caloric intake (McNab, 1978a). In seasonal deciduous forests the quality and availability of leaves fluctuates throughout the year, with young, protein-rich (high-quality) leaves with low fibre in the wet season and mature, protein-poor (low-quality) leaves with high fibre in the dry season. Leaf quality increases also within a day and is highest in the afternoon (highest protein and sugar content, but also tannin concentrations; Coley, 1983; Ganzhorn and Wright, 1994), advantageous for a species that starts feeding at the beginning of the night. *Lepilemur edwardsi* discriminates against condensed tannins, but chooses leaves with a higher alkaloid concentration. Moreover, *L. edwardsi* eats leaves with a lower protein content in comparison to the sympatric-living lemur species *Avahi occidentalis*, potentially as a result of niche-partitioning (Ganzhorn, 1988; Thalmann, 2001). Since PSM concentrations increase with rising T_a_, and simultaneously protein content decreases (Dearing, 2013; Beale *et al.*, 2018), it seems possible that *L. edwardsi* needs to increase its food intake, which in turn implicates an increase in RMR to provide for energetically expensive detoxification, at the expense of investing energy in other activities, such as territoriality or growth. As detoxification and protein turnover generate heat (Berry *et al.*, 1985; Wang *et al.*, 2010), the inability to dissipate this additional heat sufficiently at high T_a_ could be an additional factor necessitating *L. edwardsi* to resort to low-quality leaves. Furthermore, renal excretion of PSMs requires water (Foley *et al.*, 1995) and *L. edwardsi* has to budget with this resource, as it covers its water intake solely through its food; and rising T_a_, particularly unanticipated ones due to climatic changes, could result in a critically insufficient water intake of *L. edwardsi*. To compensate the lower food quality and still meet sufficient nutritional and water intake, especially during the late dry season, *L. edwardsi* presumably has to eat more, which demands a higher foraging effort to satisfy the higher DREE (Coley, 1983; Ganzhorn, 1995; Beale *et al.*, 2018). In order to meet their nutritional requirements when feeding on an unbalanced diet, which is probably the case for *L. edwardsi* in the dry season, individuals are presumably forced to overeat PSMs to balance undereating of some potentially necessary nutrients or vitamins (Simpson and Raubenheimer, 1997, 2001; Felton *et al.*, 2009), resulting in a higher need for detoxification and therefore higher RMR. This seems counterintuitive, but *L. edwardsi* might compensate the need of higher RMR for detoxification by a reduction in, e.g. activities such as travelling, thereby reducing the total amount of energy that is expended. Since *L. edwardsi* loses weight during the dry season, however, it seems that this offset is not completely met. Shifting its activity and digestion to the second half of the night when T_a_ is lower (Warren and Crompton, 1997) firstly lowers the PSM intake for *L. edwardsi* as these concentrations decrease over the course of the night and secondly could be important for detoxification, as high T_a_ has a negative influence on liver clearance function (Dearing, 2013; Beale *et al.*, 2018), potentially due to a decrease in hepatic enzyme activity and gene expression, resulting in a lower capacity for detoxification (Kurnath and Dearing, 2013). Hence, the high fluctuations of T_skin_ in *L. edwardsi* even during its activity phase could mirror different needs for detoxification and therefore enhanced liver function of this species during the night. Particularly, in the late dry season individuals seem to follow T_forest_ resp. T_treehole_ passively as T_skin_ fluctuates strongly between 28°C and 40°C (Fig. 2). Maximal T_skin_, on the other hand, was higher during the late dry season and daily fluctuation became more pronounced during the dry season. This indicates a higher heat production during this season, presumably due to the increasing need for detoxification. Another possible explanation for the higher maximal T_skin_ during the dry season could be the higher daily T_a_ during this season and therefore higher amplitudes of T_skin_. In the early mornings of the dry season, we sometimes found individuals sitting in tree crowns and outside of their tree holes basking in the sun (personal observation). They obviously use solar radiation to passively (and energetically efficiently) support endogenous thermoregulation (Fig. 2). This might enable a lowered level of metabolism, which in turn will help reduce water loss when T_a_ is high (Mitchell *et al.*, 2018; Fuller *et al.*, 2021). However, small endotherms that meet their water requirements solely through their food are unlikely to maintain continuous evaporative cooling, as they often do not have access to free water (Mitchell *et al.*, 2018), as it is the case in *L. edwardsi* during the dry season.

Many herbivores and especially arboreal folivores cover their water requirements exclusively through their food. Water limitations can even influence the distribution of arboreal folivores, as forest canopies provide only few sufficient sources of free water (Krockenberger *et al.*, 2012). When folivores reduce their food intake to avoid PSM intake and therefore costly and heat producing detoxification, they occasionally cannot cover their water requirements. This is known to be the case in the koala (*Phasocolarctos cinereus*): this species uses evaporative cooling for heat dissipation by panting, but also by licking its forearms (Degabriele *et al.*, 1978; Degabriele and Dawson, 1979). By reducing food intake to avoid detoxification, koalas may not ingest enough water to use these heat loss strategies and suffer hyperthermia (Beale *et al.*, 2018) forcing this species to climb down from their trees and search for free water, e.g. in urban areas (Gordon *et al.*, 1988; Lunney *et al.*, 2012).

These interrelations of herbivorous diet, T_a_ and water and energy budgets might become problematic with the ongoing increase of global T_a_, as well as simultaneously extended periods of droughts and higher frequency of heat waves (IPCC, 2019). Folivorous species have to spend more energy on thermoregulation and water conservation, which will ultimately threaten their reproductive success (Beale *et al.*, 2018).

## Conclusion

Despite its generally extremely low RMR and therefore low energetic demands on its habitat, *L. edwardsi* presumably has only very little scope to counter impacts due to global warming, especially towards the end of the dry season, when conditions are already challenging. Similar to its sister species *L. petteri*, *L. edwardsi* seem to struggle with the effects of environmental conditions during the dry season on food and water availability and food quality, particularly towards the end of the dry season: both show increased DREE, presumably due to higher detoxification efforts (Bethge *et al.*, 2017). Consequently, this seems to be a general physiological trait of sportive lemurs, as *L. edwardsi* and *L. petteri* show the same physiological response to seasonality and their diet. Moreover, anthropogenically induced, further increase of T_a_ might also cause an increase of PSM concentrations and a decrease of protein content in the already low-quality folivorous diet of *L. edwardsi*, while simultaneously decreasing the functionality of the liver and increasing the water loss due to heat stress. This may result in additional thermoregulatory challenges and increased energy requirements, ultimately resulting in a very small scope to deal with changing environmental conditions.

## Author contributions

Conceptualization: J.B., K.H.D.; Methodology: J.B., J.C.R., A.W., K.H.D.; Formal analysis: J.B.; Investigation: J.B.; Resources: J.B., K.H.D.; Data curation: J.B.; Writing (original draft): J.B.; Writing (review and editing): J.B., J.C.R., A.W., K.H.D.; Visualization: J.B.; Supervision: K.H.D.; Project administration: J.B.; Funding acquisition: J.B., K.H.D.

## Conflict of Interest

The authors declare no competing and financial interests.

## Supplementary Material

supp_coab075
